# A comparison of post-saccadic oscillations in European-Born and China-Born British University Undergraduates

**DOI:** 10.1371/journal.pone.0229177

**Published:** 2020-02-25

**Authors:** Diako Mardanbegi, Thomas D. W. Wilcockson, Rebecca Killick, Baiqiang Xia, Hans Gellersen, Peter Sawyer, Trevor J. Crawford

**Affiliations:** 1 School of Computing and Communications, Lancaster University, Lancaster, United Kingdom; 2 School of Sport, Exercise, and Health Science, Loughborough University, Loughborough, United Kingdom; 3 Department of Psychology, Lancaster University, Lancaster, United Kingdom; 4 Department of Mathematics and Statistics, Lancaster University, Lancaster, United Kingdom; 5 School Engineering and Applied Science, Aston University, Birmingham, United Kingdom; University of Tübingen, GERMANY

## Abstract

Previous research has revealed that people from different genetic, racial, biological, and/or cultural backgrounds may display fundamental differences in eye-tracking behavior. These differences may have a cognitive origin or they may be at a lower level within the neurophysiology of the oculomotor network, or they may be related to environment factors. In this paper we investigated one of the physiological aspects of eye movements known as post-saccadic oscillations and we show that this type of eye movement is very different between two different populations. We compared the post-saccadic oscillations recorded by a video-based eye tracker between two groups of participants: European-born and Chinese-born British students. We recorded eye movements from a group of 42 Caucasians defined as White British or White Europeans and 52 Chinese-born participants all with ages ranging from 18 to 36 during a prosaccade task. The post-saccadic oscillations were extracted from the gaze data which was compared between the two groups in terms of their first overshoot and undershoot. The results revealed that the shape of the post-saccadic oscillations varied significantly between the two groups which may indicate a difference in a multitude of genetic, cultural, physiologic, anatomical or environmental factors. We further show that the differences in the post-saccadic oscillations could influence the oculomotor characteristics such as saccade duration. We conclude that genetic, racial, biological, and/or cultural differences can affect the morphology of the eye movement data recorded and should be considered when studying eye movements and oculomotor fixation and saccadic behaviors.

## Introduction

With the emergence of the field of ‘cultural neuroscience’ new insights are emerging on the potential influence of cross-cultural factors on a wide range of measures of cognitive and low level behaviours. Whilst it is has been claimed that culture leads to differences in top-down executive functions [1, 2], there is relative paucity of work on low level behavioural measures. Previous research has found a number of eye-tracking differences between different cultures. For example, it has been observed that there are differences between groups when thinking about the answers to questions; Canadians and Trinidadians tended to look up, whereas Japanese looked down more frequently [3]. Eastern Asian participants were observed to deploy a central fixation strategy across different visual categories [4]. In general, visual attention research has identified East-West differences associated with holistic versus analytic perception and reasoning strategies [5–7]. Westerners tend to fixate more often, more quickly and more accurately on focal objects [7–10] compared to Easterners. In contrast, Easterners allocate attention more globally and broadly in visual processing compared to Westerners [7, 8]. Easterners are found to make more numerous [11, 12] and shorter duration [11] fixations, and consume longer searching time in visual searching tasks [12]. Whether these differences between different groups are due to nature (biology, genetics, race) or nurture (culture) is still debated. Few studies have investigated group differences at the level of the brainstem neural control signals, the oculomotor plant and physiology of the eye structures. It is not precisely clear at this point what the origin or fundamental nature of post-saccadic oscillations (PSO) is at this time [13]. They may reflect some lower level oculomotor control signals from the brain, or they may reflect artifacts of the recording technique and they may be influenced by a number of factors (e.g. cultural, genetic, and neurophysiologic factors, as well as, neuroanatomical differences environmental factors). The principal aim of this work was to investigate whether there are group differences in PSO form. We cannot determine the cause of any differences note at this time. We hope through this paper to develop a greater understanding of saccadic eye movements and the factors which may affect the saccade-related-metrics (e.g., saccade duration). Such research may have implications for administration of saccadic eye movement tasks on different cultures.

There is an activate debate about the cultural influence in visual attention. Remarkably, nurture has been reported to be more influential in shaping human oculomotor behavior than nature [14]. However, in the work of *Rayner et al*. [15], no difference was found in scene perception between Eastern and Western Viewers. Differences in cognition and perceptual processes have been observed by eye movement research on Chinese and Caucasian participants (e.g. [10]). For example, effects of culture on the different aspects of visual attention have been observed for fixation duration, number of fixations, and saccades [10, 16]. Also, more recently, Knox and Wolohan (2014) suggest a distinction in oculomotor phenotype between Chinese and Caucasian as their British-Chinese participants performed analogously to Chinese participants from China [2]. This, suggests that environmental factors cannot be the critical explanatory factor for the eye movement differences.

Nystrom, Hooge, and Holmqvist (2013) compared the motion of the pupil center and the eyeball (measured through the center of limbus) in a video based eye-tracker and observed that the post-saccadic oscillations (PSO) of the pupil do not necessarily match the oscillations of the eyeball [13]. Subsequently, they showed how this can affect the pupil and corneal reflection signals measured by the eye tracker [17]. Their results indicate that more knowledge about PSOs is essential to fully understand the underlying cause of this phenomenon and to compare the findings obtained from video-based eye trackers with other eye tracking technologies. They suggested that while the eye tracking technique could have a significant affect on the measurement of the post-saccadic oscillations, PSOs may also differ between populations. Other studies showed the effect of pupil size and saccade peak velocity (and saccade amplitude accordingly) on the shape of the PSO signals [18, 19]. Mardanbegi et al. (2017) observed an aging effect on PSO; increased PSO was linearly associated with age [20]. However, could differences in PSO be observed between age-matched cross cultural, cross racial, or cross genetic populations?

In this study, we looked at the eye movements of two groups of participants (European-Born and China-Born British University Undergraduates) recorded in a video watching experiment. We extracted the post-saccadic oscillations from the eye tracking data and compared these oscillation characteristics across the two groups. The results show that the shape of the PSOs were significantly different between the two groups. The differences in PSO are important to consider when studying eye movements of different groups of people as it may have methodological implications for measurement of eye movement metrics. Further, our results may enable us to better understand the origin of the PSOs and increasing the knowlegede about whether cultural, genetic, neurophysiologic, or oculomotor factors could affect PSO form.

## Materials and methods

The eye tracking data was recorded in a video watching experiment where participants viewed three videos which were each displayed for 40 seconds. The videos were (1) Coronation of the Queen Elizabeth II, (2) Gordon Brown and family leaving Downing Street after losing the general election in 2010, and (3) Neil Armstrong landing on the moon in 1969. Participants were given a general introduction before each video about the content of that video, but they were informed that they could freely view each video on the first viewing. On the second and third viewing the participants were asked questions designed to encourage visual search of each video. These video watching tasks enabled us to obtain PSO signals for a wide range of saccadic eye movements with different amplitudes collected from naturalistic viewing conditions more equivalent to that in the real world.

### 0.1 Participants and apparatus

Our dataset included 94 participants: 42 European-born (Caucasians) students with ages ranging from 18 to 36 (mean = 21.0, SD:3.46)(9 male and 33 female), and 52 China-born (Chinese) students with ages ranging from 19 to 36 (mean = 23.77, SD:2.64)(25 male and 27 female).

All participants were undergraduate students recruited from a British university. Written informed consent was obtained and the study was approved by Lancaster University ethics committee and also the National Research Ethics Service (Health Research Authority (HRA), 11/NW/0723). All of the Chinese participants were born and raised in China and had moved to UK to undertake their undergraduate studies. Caucasian participants (except four who where born in mainland Europe) were born and raised in the UK and were all attending the same British university at the time of testing.

Potential participants were made aware prior to the study that the study involved eye movement measurement. Participants were asked to report any related medical history. None of the participants were using any medications.

A fixed-head setup using an Eyelink 1000 eye tracking system (SR Research Ltd., Ontario, Canada) was used to record participants’ dominant eye (determined using the Miles test [21] and tracked accordingly) at 500 Hz. A chin-rest with a forehead support was used to help the subjects to keep their head still during the experiment. Participants were seated 55 cm away from a 24-inch Dell monitor (with the resolution of 1024 × 768 pixels and refresh rate of 60 Hz) during the data collection. The camera was positioned horizontally to ensure that the camera was directly facing the participants’ tracked eye and the eye appeared in the center of the experimenter’s display monitor. A single user calibration with 9 points was performed prior to the experiment. The result of the calibration was assessed by doing a validation test using 9 points immediately after the calibration. The calibration was repeated when the result of the validation reported by the eye tracker was poor.

### 0.2 Procedure and data collection

In the video watching experiment, the participants viewed three videos which were each displayed for 40 seconds. The videos were (1) Coronation of the Queen Elizabeth II, (2) Gordon Brown and family leaving Downing Street after losing the general election in 2010, and (3) Neil Armstrong landing on the moon. Participants were given a general introduction before each video about the content of that video. Each participant performed a free viewing task followed by two more instructed tasks in which they were asked to find answers to questions designed to direct the top-down control of eye gaze (e.g. Question 1 of Video 3 was “How many bald men are in the room?”) and to encourage visual search of each video. The two questions for each video were the same for all participants. The eye movements were collected from 9 video trials per participant. Each video lasted 40 seconds. The eye tracking data provided us with a wide range of saccadic eye movements from which we could extract the PSO signals.

## Data pre-processing

Saccade detection was done in the Eyelink Dataviewer software. We filtered those saccades that had a duration larger than 200 ms or a peak velocity of larger than 500 deg/sec which were considered as outliers. We also filtered those with amplitude larger than 20 deg or smaller than 1.5 deg because we didn’t want to include microsaccades or unexpected large saccades in our PSO analysis. We used the PSOVIS software [22] to extract and align the PSO signals from the eye movement data. The PSOVIS software made it possible to include, align, and compare all saccades in the analysis regardless of their saccade amplitude and direction.

**Fig 1:**
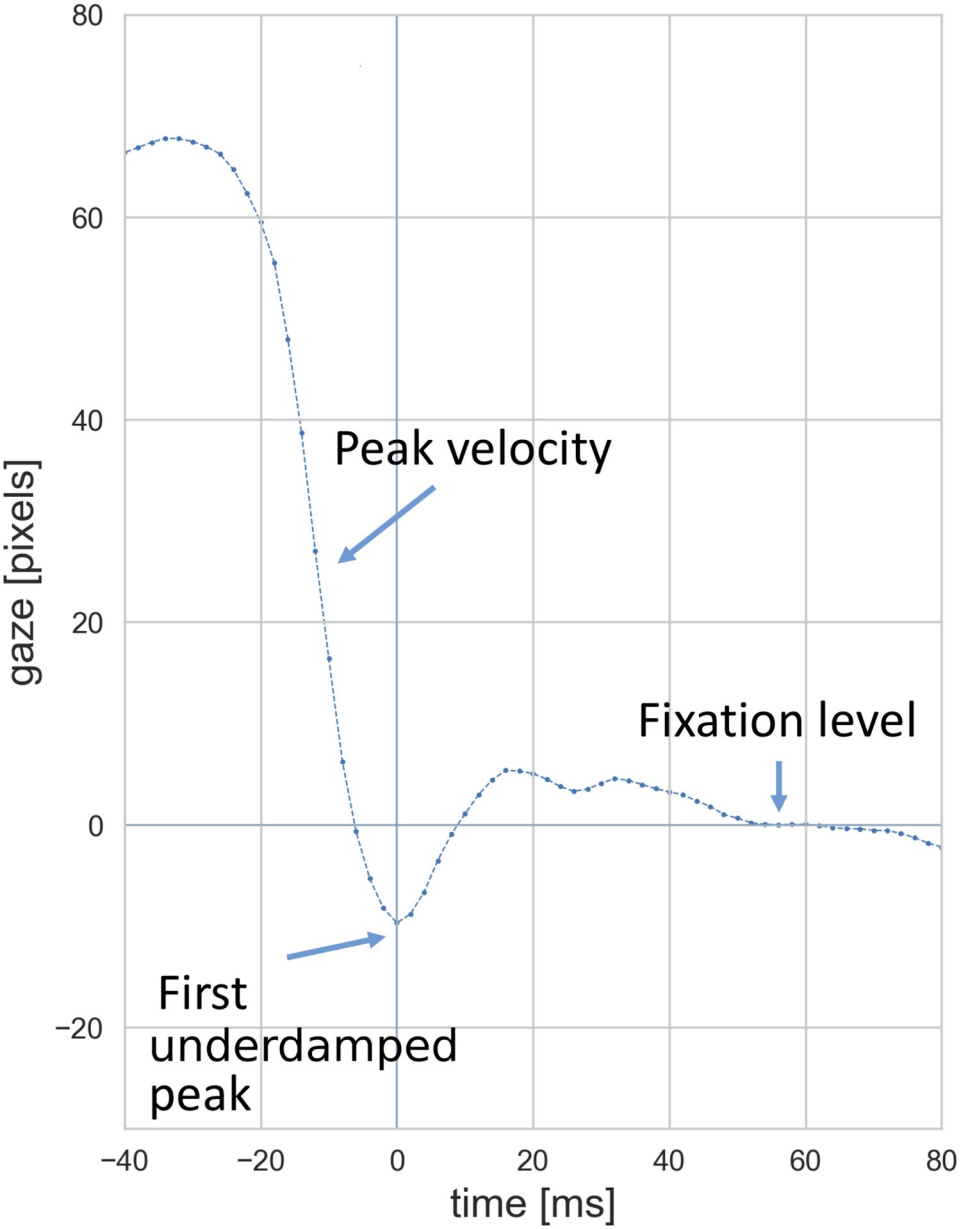
An example saccade and the PSO. All PSOs were aligned temporally relative to their first overshoot peak and spatially with respect to their fixation position.

The PSO signals are represented by *PSO* = *S*(*t*) where t is measured relative to the time where the first critical point (zero velocity) of the saccade happens after the maximum velocity. *S* represents the gaze coordinate along the direction of the saccade (e.g., x coordinate of the gaze in a horizontal saccade). *t* = 0 is set to the time where the first overshoot peak of the signal happens, therefore, all the PSO signals are aligned temporally relative to *t* = 0 (Fig 1). The fixation level after each saccade is defined by averaging the gaze values within the time window of t = 40 ms to t = 70 ms which is a period of 30 ms starting 40 ms after the first overshoot. This was to ensure that the oscillations had terminated before estimation of the fixation positions. The PSO was observed in the majority of the saccades and over 90% of the saccades had an overshoot. All PSO signals were spatially aligned with respect to their fixation level *S* = 0. Multiple PSOs were combined into one signal by taking the median of all values at each time step ($\underset{i=1,2,...,n}{\text{median}}\left\{S{\left(t\right)}_{i}\right\}$ where *n* is the number of signals).

## Results

As an overall comparison of the data quality between the two groups, we extracted the details of the validation step stored as EDF files generated by the EyeLink tracker and compared the calibration quality between the two groups. The mean of the average error (*err*) measured in degrees of visual angle is shown in Table 1. Other general oculomotor measures such as the mean of the fixation count (*N*_*fix*_) and saccade count (*N*_*sac*_) per subject and the mean of the fixation duration (*dur*_*fix*_) are presented in Table 1 for both groups. The number of saccades was counted after the filtering process described above. In total 25072 saccades were obtained from the Caucasian group and 29189 from the Chinese group. We found no statistical differences between these measures across the groups.

**Table 1:** General oculomotor statistics for the data collected from the two groups.

	*err*	*N*_*sac*_	*N*_*fix*_	*dur*_*fix*_
Caucasians	0.42°(SD = 0.21)	860.5(SD = 161.1)	971.5(SD = 170.7)	377.1 ms(SD = 363.1)
Chinese	0.48°(SD = 0.14)	788.1(SD = 147.2)	927.2(SD = 170.5)	377.7 ms(SD = 367.4)

Values shown inside parenthesis are the standard deviations. *err*: mean of the average error, *N*_*sac*_: average number of saccades per subject, *N*_*fix*_: average number of fixations per subject, *dur*_*fix*_: mean of the fixation duration.

**Fig 2:**
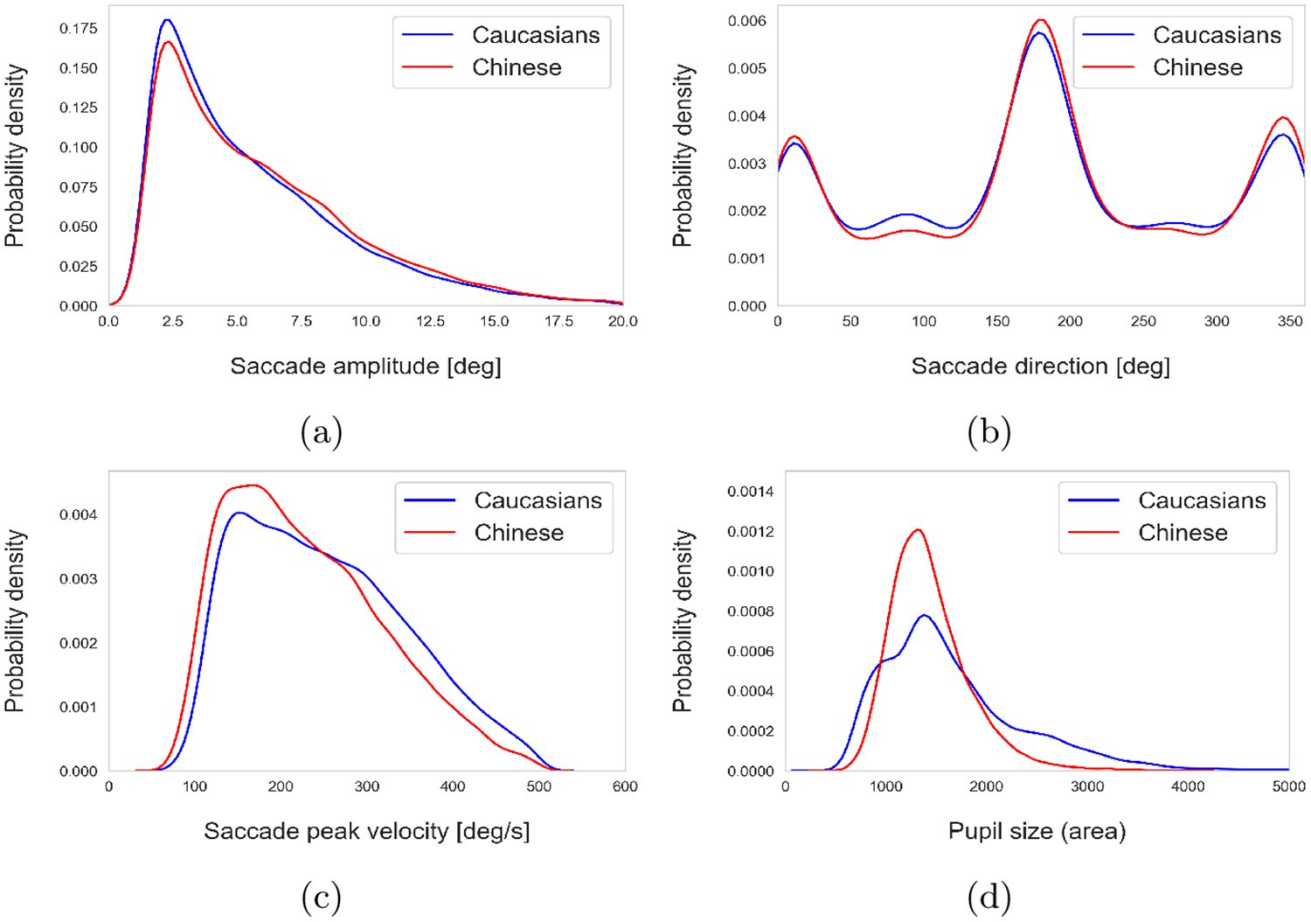
Kernel density of various measurements across the two groups.

Fig 2 shows the distribution of various oculomotor measures indicating that those measurements except for pupil size were relatively similar across the two groups of participants. We therefore consider the pupil size as an independent factor later in our analysis.

**Fig 3:**
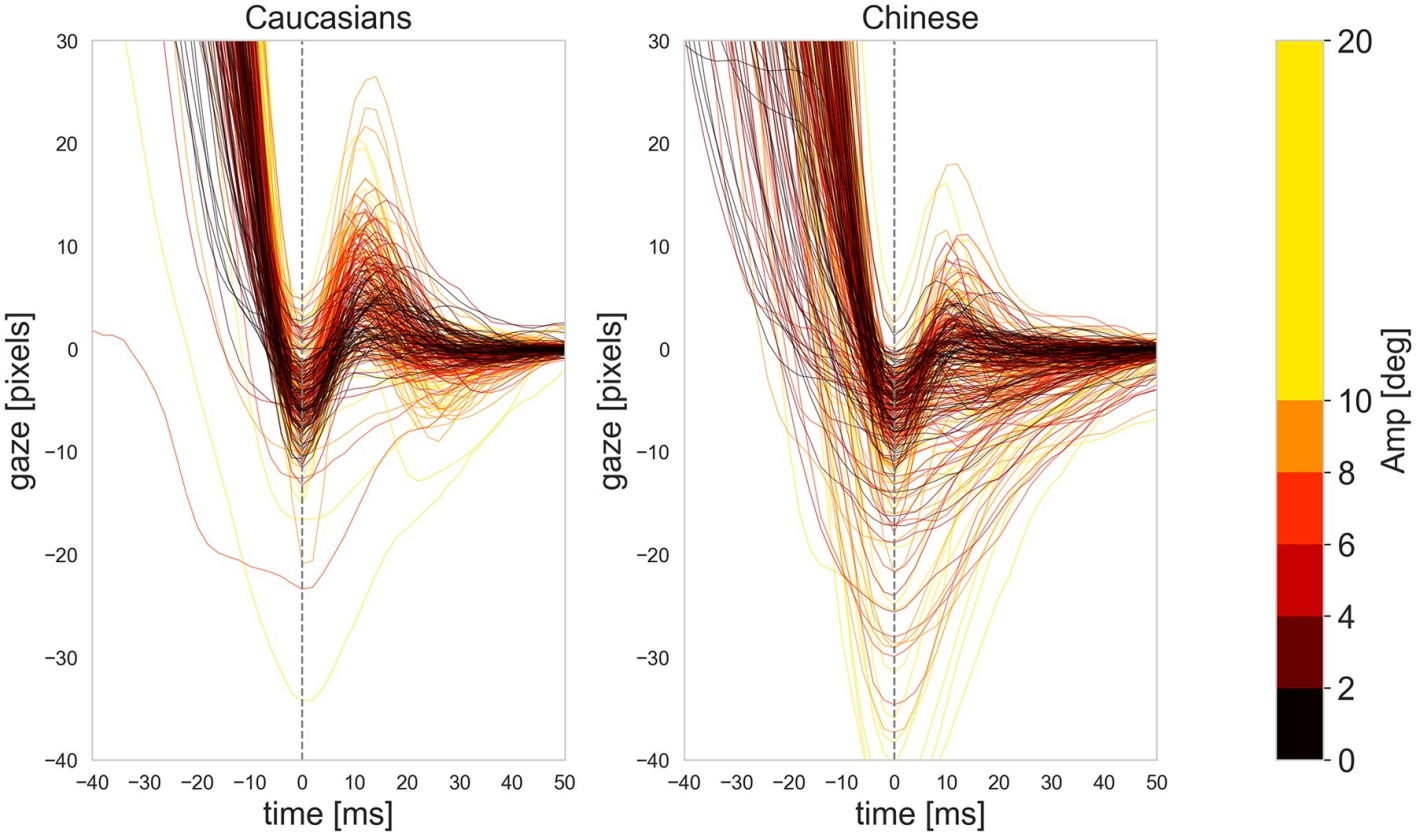
The median PSO signals of all the saccades for Caucasians and Chinese groups. Each signal shows the median of all recorded signals per subject inside each range of saccade amplitude. Colors represent the amplitude for each PSO.

Fig 3 shows the PSO signals for 6 different ranges of saccade amplitude from 0 to 20°. To avoid crowding the figure, we only show the median signal within each range instead of individual signals. PSO signals are colored differently for different saccade amplitudes. Each median signal in the figure represents the median of all PSOs of an individual subject that belong to saccades with amplitudes within a certain range. In order to compare the signals between our two groups we measured two features from each signal. The first feature is the first overshoot peak of the signals that happens at *t* = 0 (*S*(0)). The second feature was the PSO value at *t* = 10 (*S*(10)) where the first undershoot of the majority of the PSOs happen.

A typical approach for analyzing data of this type (several observations from each subject and several subjects) is to fit a Linear Mixed Model (LMM) [23]. Previous research [20] has considered the difference between groups using a single point of the PSO signal (*t* = 0). Whilst this is informative, we are interested in the joint difference between the PSO at *t* = 0 and at *t* = 10. Thus, for each saccade, we have a bivariate response variable. In building our bivariate model we considered the variables: pupil size, age, gender, saccade amplitude and group. We didn’t include saccade peak velocity as an extra variable because any possible effect of peak velocity on the PSO is indirectly exerted via saccade amplitude due to the main sequence relationship between saccade peak velocity and amplitude. The most (statistically) appropriate mixed model is given in Eq 1 where square root of PSO value at t = 0 and t = 10 for person *j* is a function of square root of pupil size (P), logarithm of the saccade amplitude (A) and group. The *u*_*j*_ term is the additional term allowing a different intercept for each person. Interestingly age is not significant in our analysis, this is likely due to the lack of variability in age, with 90% of our saccades being taken from participants 18-25.

$$\begin{array}{c} (\begin{array}{c} \sqrt{S{\left(0\right)}_{j}}\\ \sqrt{S{\left(10\right)}_{j}} \end{array})=(\begin{array}{c} \beta_{1}^{0}\\ \beta_{1}^{10} \end{array})+(\begin{array}{c} \beta_{2}^{0}\\ \beta_{2}^{10} \end{array})\sqrt{P}+(\begin{array}{c} \beta_{3}^{0}\\ \beta_{3}^{10} \end{array})\log A+(\begin{array}{c} \beta_{4}^{0}\\ \beta_{4}^{10} \end{array})I_{Chinese}+u_{j}. \end{array}$$ (1)

**Fig 4:**
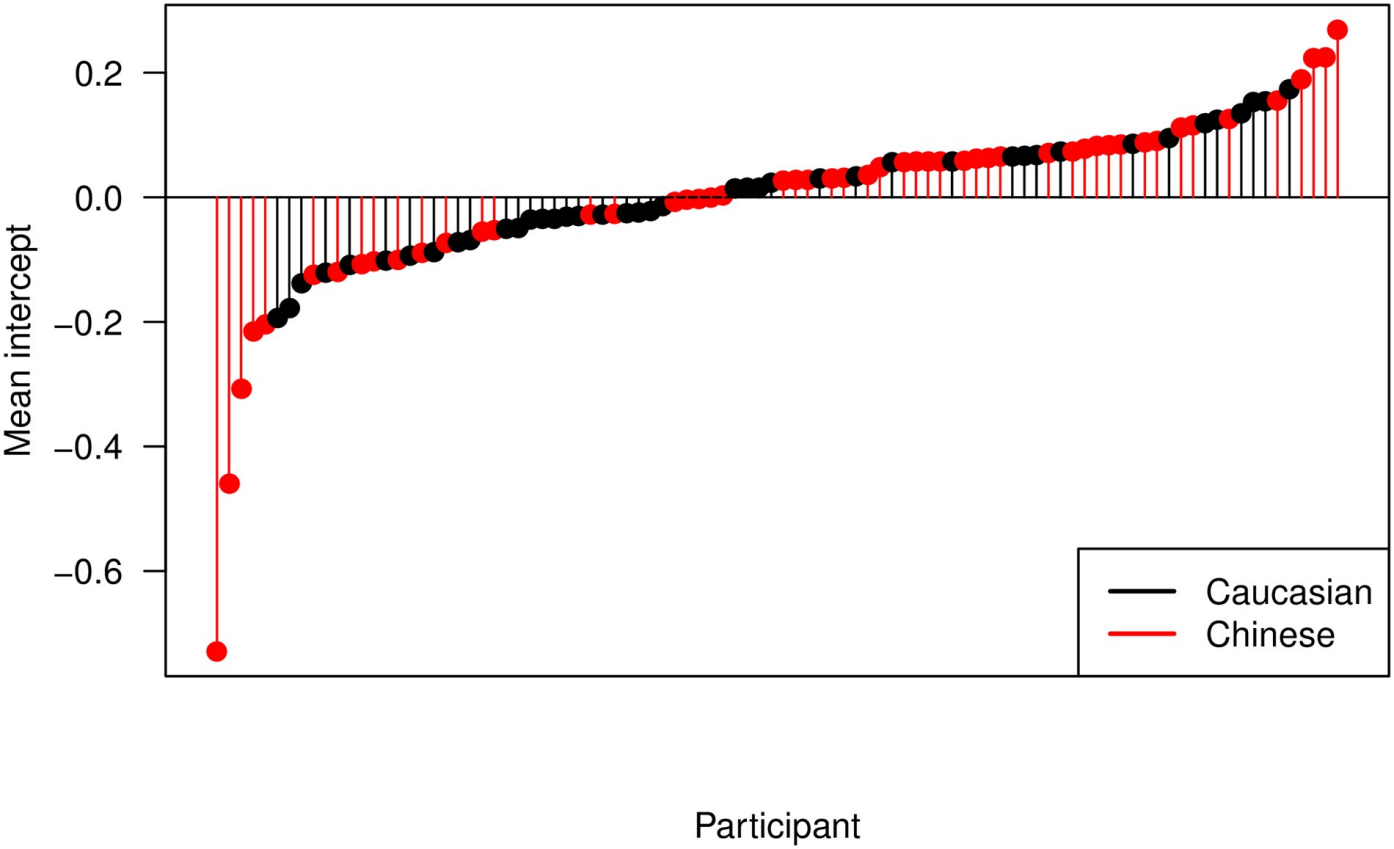
Mean random effect intercepts per participant accompanying the estimates of the fixed effects from Table 2.

The Linear Mixed Effects model detailed in Eq 1 was fit using the lmer function from the lme4 [24] package in the statistical software R [25]. The values of the fitted mixed effects model are given in Table 2 and Fig 4. This demonstrates that the PSO amplitude at *t* = 0 for a typical person in the Chinese group is 0.0877 times lower than that of a typical person in the Caucasian group. The effect is even more pronounced for the PSO amplitude at *t* = 10 where a typical person in the Chinese group is -0.2802 times lower. Recall this is on the square root scale. Interestingly we also see that the saccade amplitude has a negative effect at *t* = 0 and a positive effect at *t* = 10. This is expected as the amplitude of the saccade affects the depth at *t* = 0 and the height at *t* = 10.

**Table 2:** Results of the bivariate linear mixed model analysis. Each of the variables in our final model alongside its estimate and 95% confidence interval.

	Estimate	CI		Estimate	CI	
	(t = 0)	2.5%	97.5%	(t = 10)	2.5%	97.5%
Intercept	10.727	10.678	10.777	8.912	8.863	8.962
$\sqrt{P}$	-0.0053	-0.0059	-0.0046	-0.0007	-0.0012	-0.0001
log*A*	-0.0166	-0.0207	-0.0125	0.1019	0.0962	0.1077
*I*_*Chinese*_	-0.0877	-0.1437	-0.0318	-0.2802	-0.2875	-0.2729

## Further investigations

Because the groups were not balanced in terms of gender, it may be that the difference in the PSOs of the two groups are caused by the difference in the number of female participants in the Chinese group. Gender was balanced in the Chinese group where 51.92% of the population were male and 48.08% female. We further looked at the effect of gender on the PSO signals. We also investigated whether wearing glasses could affect the size of the PSO signals. Horizontal and vertical saccades were also compared in terms of their PSO.

### Effect of gender

**Fig 5:**
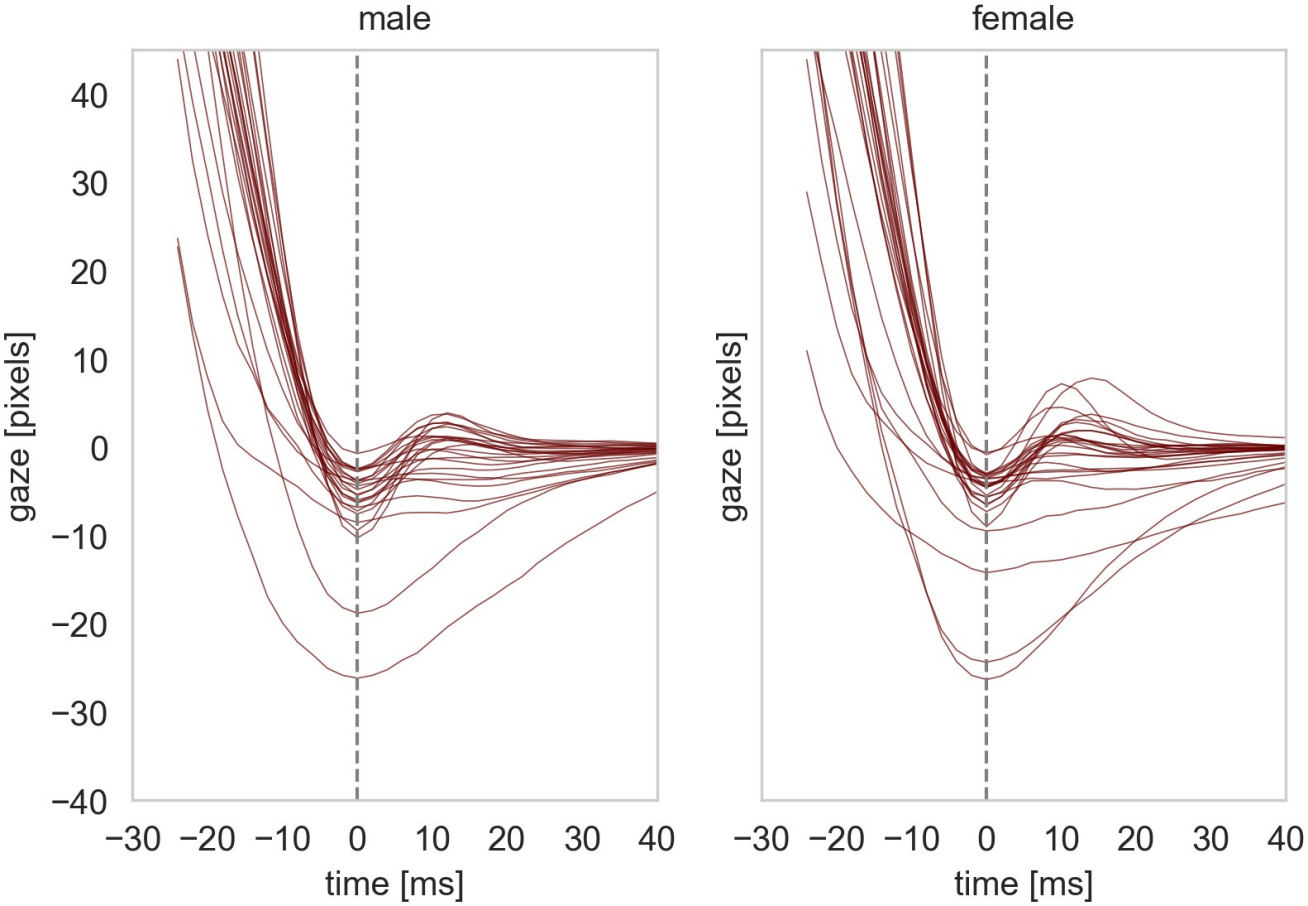
The median PSO signals of male and female participants of the Chinese group.

We divided the Chinese group into two subgroups (male and female) and compared the PSO signals between these groups. Fig 5 shows the PSO signals of the 27 male and 25 female subjects in the Chinese group for different ranges of saccade peak velocities. The results show that the same pattern with very high under-damped oscillations for higher ranges of peak velocities are visible in both genders and it unlikely that the difference between the Caucasians and Chinese groups is coming from the gender differences.

### Effect of saccade direction

While the distribution of the saccade direction was the same in the Caucasians and Chinese groups (as seen in Fig 2), vertical and horizontal saccades were compared in terms of PSO and we found no significant difference between the PSO signals of the vertical and horizontal saccades in either of the groups.

### Effect of wearing glasses

In our study, we didn’t record information about whether subjects were wearing glasses. However, before each recording, participants were asked to remove their glasses if possible as it aids the calibration process. Wearing glasses may change the appearance of the pupil in the eye image and change the shape of the oscillations of pupil center (and the glint) measured by the tracker. However, in our study, PSO signals were not directly extracted from the pupil center instead they were extracted from the gaze data obtained from pupil center and corneal reflection which represent a point in the screen coordinate system. In this case, wearing glasses should not have a significant effect on the amplitude of the PSO, because the total amplitude of the saccade (obtained from the gaze data) will be the same with and without glasses when the eye moves between two arbitrary points (A and B). We further tested this on a person wearing thick glasses (SPH = -1.75, CYL = -4.25 and AXIS = 180) performing a pro-saccade task with and without glasses. We found no significant differences between the size and the shape of the PSO signals of the two conditions (50 signals were recorded per condition).

## Discussion

The results of our study show that Chinese and Caucasian students studying in Britain have different PSO characteristics. Our results provide no information about the cause or basis of these differences. Knox and Wolohan, 2014, suggest a distinction in oculomotor phenotype between Chinese and Caucasian [2], which suggests there could also be structural aspects of the iris which differ between populations that affect PSO. However, Amatya, Gong, and Knox, 2011 suggest any differences between populations may be the result of top-down executive functions [1]. A resolution of the question of whether Chinese-Caucasian PSO differences are due to nature or nurture is beyond the scope of the current paper. However, we hope that the data provided here could provide new evidence about the origin of PSO.

**Fig 6:**
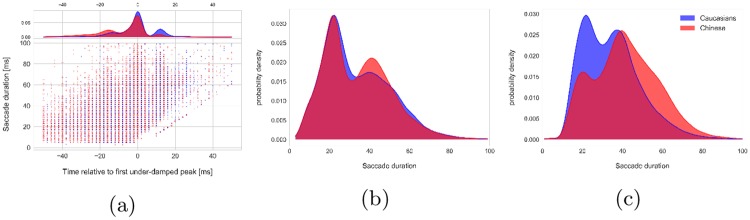
Distribution of the saccade endings relative to the first under-damped peak(a), kernel density estimates of saccade duration based on saccade endings detected by the EyeLink software (*SE*_*eyelink*_) (b), kernel density estimates of saccade duration based on saccade endings at the first under-damped peak (*SE*_*t*=0_) (c). The Chinese and the Caucasians data are respectively represented by red and blue colors.

**Fig 7:**
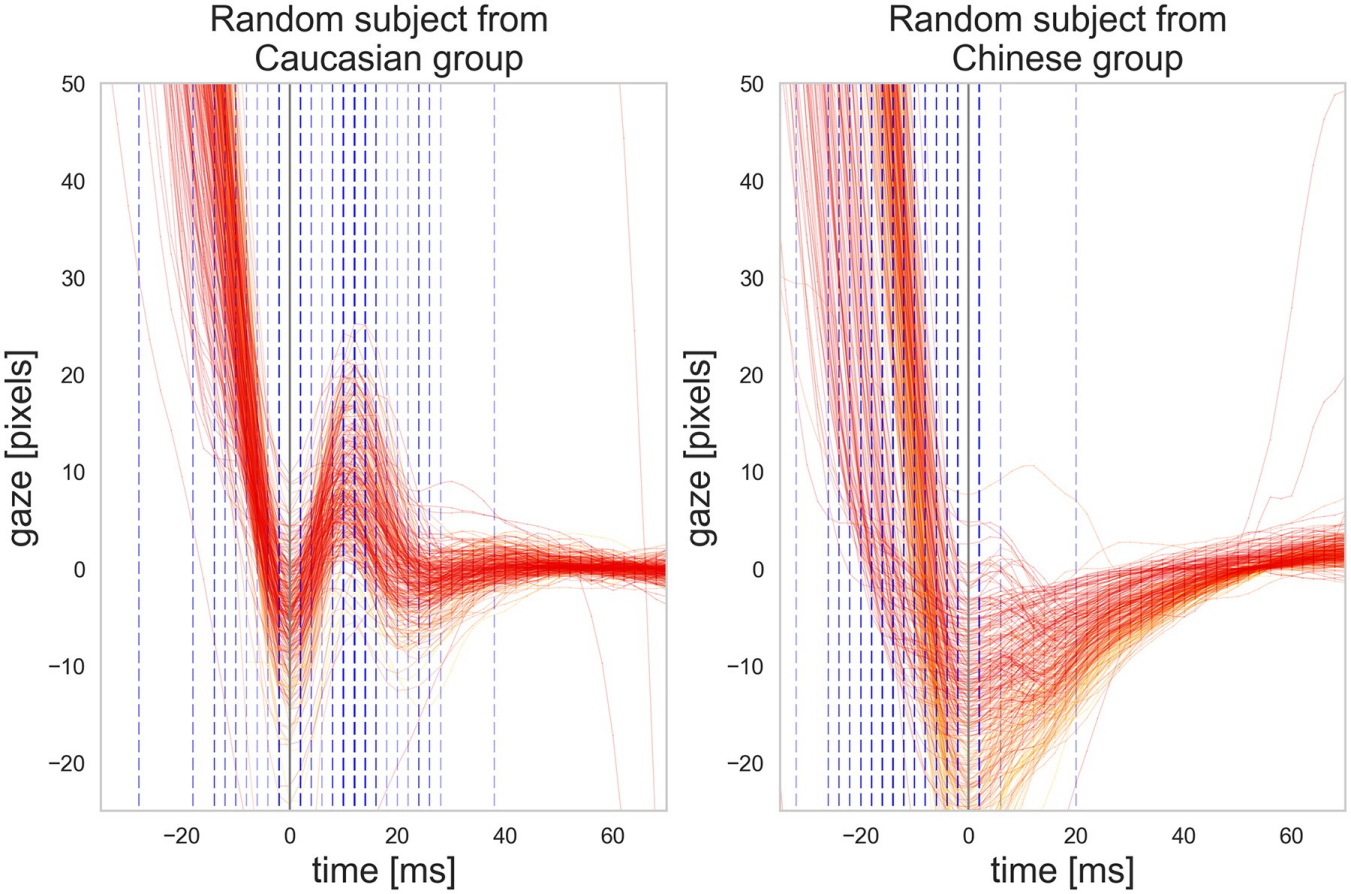
All individual PSO signals for two randomly chosen participants from each group. The vertical dashed lines indicate the end of each saccade as detected by the EyeLink software.

As pointed out by Nyström and Holmqvist, 2010 [26], PSOs are treated differently across different oculomotor event detection algorithms and because fixations are defined vaguely and implicitly in the literature [27], event detection algorithms may assign PSOs to the saccades or merge them with the fixations. This means that depending on the event detection algorithm used, differences in the PSOs between two groups may yield different eye movement measures such as fixation and saccade duration between the two groups. To see how much the saccade detection algorithm used by the EyeLink software has been affected by the PSO differences of the two groups, we looked at the saccade offsets as detected by the EyeLink software (*SE*_*eyelink*_) in relation to the first under-damped peak of the signals (*S*(0)). Fig 6a shows how saccade offsets are distributed around the time 0 where the first critical point of the saccade happens after the maximum velocity. While more than 60% of the saccade offsets detected by the EyeLink software were within the range of [−10*ms*, 10*ms*] around the first under-damped peak, distribution of the saccade offsets around this time window differs in our two groups. This could be attributed to the difference in the PSO signals between the two groups. As we see in Fig 6a, there were many saccade offsets detected around 15 ms in the Caucasian group which is perhaps because of the changes in the saccade velocity around the second bump of the PSO. There were also more saccade offsets detected before time 0 in the Chinese group than in the Caucasian group. Fig 7 shows the PSO signals of two randomly chosen participants from each group as well as the saccade offsets indicated by vertical dashed lines.

The differences in the saccade detection between the two groups could result in the differences in the saccade duration. The higher number of saccade offsets detected before *t* = 0 in the Chinese group will reduce the average saccade duration for this group. Fig 6b shows the distribution of the saccade duration measured based on the saccade offsets detected by the EyeLink software *SE*_*eyelink*_.

We saw no significant difference between the saccade durations of the two groups even though lower durations were expected for the Chinese group. In order to make the saccade offsets independent of the shape of the PSOs, we assumed that every saccade ends at *t* = 0 (referred to as *SE*_*t*=0_), and we measured the saccade durations using the new endings (results shown in Fig 6c). By comparing the results with Fig 6b, it is clear that the EyeLink event detection algorithm has underestimated the saccade duration of the Chinese group.

Based on our findings, the size of the PSO signals are larger in the Chinese group for higher peak velocities. The oscillation of the pupil, relative to the eye at the end of each saccade presumably causes the visual input to oscillate slightly. It is unclear whether the visual input differs between the two groups as a result of differences in the post-saccadic oscillations. It may be that both groups have adapted different strategies for reducing perceptual wobble induced by large and fast saccades. Cultural elements have been studied to explain this difference. The experience with a given writing system is revealed to have a large impact on fixation durations and saccade lengths [11]. For example, Chinese words are constructed by sophisticated strokes with a mono-syllable such that people need to pay more attention to details in order to recognize a word correctly, which might encourage Chinese to cultivate the habit of paying attention to details and contexts [28, 29]. This experience with different writing styles may affect PSO. However, how these between-group eye movement operate physically is unclear, our PSO signals were obtained from the gaze data and not the pupil center or iris center so it is difficult to ascertain the actual muscular source of the PSO.

## Conclusion

Post-saccadic oscillation eye movements (PSO) were compared between two different populations: university students born in China or in Europe. This study observed differences in PSO between Chinese and Caucasian participants. The differences in PSO signals were evident at different ranges of peak velocities where the size of the PSO signals were larger in the Chinese group compared to the Caucasian group.

Our view is that cultural factors at the level of shared knowledge, beliefs, practices, and values are unlikely to account for the low level and hard-wired PSO differences we have observed between the Chinese and Caucasian. A more compelling hypothesis is that these effects are a result of a combination of overlapping cultural and biological factors including diet, metabolism and biochemistry [14, 30]. The results may indicate that Caucasians and Chinese have developed or are genetically predisposed to different strategies for reducing perceptual wobble after a saccade. It may be that these differences may be an extraneous variable which may need to be considered when measuring saccadic eye movements with video-based eye trackers. Finally, this work has wider methodical implications. The differences in the PSO eye movements between two populations may affect the performance of the event detection algorithms which could result in the differences in the saccade duration between the two groups.

## References

[1] Amatya N, Gong Q, Knox PC. Differing proportions of ‘express saccade makers’ in different human populations. Experimental Brain Research. 2011;210(1):117–129. 10.1007/s00221-011-2609-z 21374077

[2] Knox PC, Wolohan FDA. Cultural Diversity and Saccade Similarities: Culture Does Not Explain Saccade Latency Differences between Chinese and Caucasian Participants. PLOS ONE. 2014;9(4):1–7. 10.1371/journal.pone.0094424 PMC3978040 24709988

[3] McCarthy A, Lee K, Itakura S, Muir DW. Cultural display rules drive eye gaze during thinking. Journal of Cross-Cultural Psychology. 2006;37(6):717–722. 10.1177/0022022106292079 19122788 PMC2613330

[4] Kelly DJ, Miellet S, Caldara R. Culture shapes eye movements for visually homogeneous objects. Frontiers in psychology. 2010;1 10.3389/fpsyg.2010.00006 21833189 PMC3153738

[5] Nisbett RE, Peng K, Choi I, Norenzayan A. Culture and systems of thought: holistic versus analytic cognition. Psychological review. 2001;108(2):291 10.1037/0033-295x.108.2.291 11381831

[6] Nisbett R. The Geography of Thought: How Asians and Westerners Think Differently… and Why. 2003.

[7] Boduroglu A, Shah P, Nisbett RE. Cultural differences in allocation of attention in visual information processing. Journal of Cross-Cultural Psychology. 2009;40(3):349–360. 10.1177/0022022108331005 20234851 PMC2838246

[8] Stoesz BM, Jakobson LS, Kilgour AR, Lewycky ST. Local processing advantage in musicians: Evidence from disembedding and constructional tasks. Music perception: An Interdisciplinary Journal. 2007;25(2):153–165. 10.1525/mp.2007.25.2.153

[9] Tan YY. East-West cultural differences in visual attention tasks: Identifying multiple mechanisms and developing a predictive model. 2016.

[10] Chua HF, Boland JE, Nisbett RE. Cultural variation in eye movements during scene perception. Proceedings of the National Academy of Sciences of the United States of America. 2005;102(35):12629–12633. 10.1073/pnas.0506162102 16116075 PMC1194960

[11] Rayner K, Li X, Williams CC, Cave KR, Well AD. Eye movements during information processing tasks: Individual differences and cultural effects. Vision research. 2007;47(21):2714–2726. 10.1016/j.visres.2007.05.007 17614113 PMC2048814

[12] Alotaibi A, Underwood G, Smith AD. Cultural differences in attention: Eye movement evidence from a comparative visual search task. Consciousness and cognition. 2017;55:254–265. 10.1016/j.concog.2017.09.002 28946046

[13] Nyström M, Hooge I, Holmqvist K. Post-saccadic oscillations in eye movement data recorded with pupil-based eye trackers reflect motion of the pupil inside the iris. Vision research. 2013;92:59–66. 10.1016/j.visres.2013.09.009 24096093

[14] Caldara R, Richoz Ar, Liu Y, Lao J. Cultural Diversity in Eye Movements is shaped by Nurture not Nature. International Journal of Psychology. 2016;51:1015.

[15] Rayner K, Castelhano MS, Yang J. Eye movements when looking at unusual/weird scenes: Are there cultural differences? Journal of Experimental Psychology: Learning, Memory, and Cognition. 2009;35(1):254 10.1037/a0013508 19210095 PMC2668126

[16] Goh JO, Chee MW, Tan JC, Venkatraman V, Hebrank A, Leshikar ED, et al Age and culture modulate object processing and object—scene binding in the ventral visual area. Cognitive, Affective, & Behavioral Neuroscience. 2007;7(1):44–52. 10.3758/CABN.7.1.44 17598734

[17] Hooge I, Holmqvist K, Nyström M. The pupil is faster than the corneal reflection (CR): Are video based pupil-CR eye trackers suitable for studying detailed dynamics of eye movements? Vision research. 2016;128:6–18. 10.1016/j.visres.2016.09.002 27656785

[18] Hooge I, Nyström M, Cornelissen T, Holmqvist K. The art of braking: Post saccadic oscillations in the eye tracker signal decrease with increasing saccade size. Vision Research. 2015;112:55–67. 10.1016/j.visres.2015.03.015 25982715

[19] Nyström M, Hooge I, Andersson R. Pupil size influences the eye-tracker signal during saccades. Vision research. 2016;121:95–103. 10.1016/j.visres.2016.01.009 26940030

[20] Mardanbegi D, Killick R, Xia B, Wilcockson T, Gellersen H, Sawyer P, et al Effect of aging on post-saccadic oscillations. Vision Research. 2018;143:1–8. 10.1016/j.visres.2017.08.006 29197475

[21] Roth HL, Lora AN, Heilman KM. Effects of monocular viewing and eye dominance on spatial attention. Brain. 2002;125(9):2023–2035. 10.1093/brain/awf210 12183348

[22] Mardanbegi D, Wilcockson T, Xia B, Gellersen H, Crawford T, Sawyer P. PSOVIS: An interactive tool for extracting post-saccadic oscillations from eye movement data. COGAIN Symposium, 19th European Conference on Eye Movements (ECEM 2017). 2017.

[23] Ga lecki A, Burzykowski T. Linear Mixed-Effects Models Using R: A Step-by-Step Approach Springer Texts in Statistics. Springer New York; 2013 Available from: https://books.google.co.uk/books?id=rbk_AAAAQBAJ.

[24] Bates D, Mächler M, Bolker B, Walker S. Fitting linear mixed-effects models using lme4. arXiv preprint arXiv:14065823. 2014.

[25] Team RC, et al. R: A language and environment for statistical computing. 2013.

[26] Nyström M, Holmqvist K. An adaptive algorithm for fixation, saccade, and glissade detection in eyetracking data. Behavior Research Methods. 2010;42(1):188–204. 10.3758/BRM.42.1.188 20160299

[27] Hooge ITC, Niehorster DC, Nyström M, Andersson R, Hessels RS. Is human classification by experienced untrained observers a gold standard in fixation detection? Behavior Research Methods. 2017. 10.3758/s13428-017-0955-x PMC7875941 29052166

[28] Tan LH, Laird AR, Li K, Fox PT. Neuroanatomical correlates of phonological processing of Chinese characters and alphabetic words: A meta-analysis. Human brain mapping. 2005;25(1):83–91. 10.1002/hbm.20134 15846817 PMC6871734

[29] Han S, Northoff G. Culture-sensitive neural substrates of human cognition: A transcultural neuroimaging approach. Nature Reviews Neuroscience. 2008;9(8):646–654. 10.1038/nrn2456 18641669

[30] Kardan O, Shneidman L, Krogh-Jespersen S, Gaskins S, Berman MG, Woodward A. Cultural and Developmental Influences on Overt Visual Attention to Videos. Scientific reports. 2017;7:11264 10.1038/s41598-017-11570-w 28900172 PMC5595807

